# Distribution of CPP-Protein Complexes in Freshly Resected Human Tissue Material

**DOI:** 10.3390/ph3030621

**Published:** 2010-03-12

**Authors:** Külliki Saar, Helgi Saar, Mats Hansen, Ülo Langel, Margus Pooga

**Affiliations:** 1 Institute of Molecular and Cell Biology, University of Tartu; Riia Street 23, 51010 Tartu, Estonia; 2 Department of Pathology, University of Tartu Hospital; Puusepa Street 8, 50411 Tartu, Estonia; 3 Department of Biochemistry, University of Tartu; Ravila Street 19, 50411 Tartu, Estonia; 4 Molecular Biotechnology Lab, Institute of Technology, University of Tartu; Nooruse Street 1, 50411 Tartu, Estonia; 5 Department of Neurochemistry, Stockholm University; Svante Arrhenius väg 21A, 10691 Stockholm, Sweden

**Keywords:** cell-penetrating peptides, *ex vivo*, tissue selectivity, carcinoma

## Abstract

Interest in cell-penetrating peptides (CPPs) as delivery agents has fuelled a large number of studies conducted on cultured cells and in mice. However, only a few studies have been devoted to the behaviour of CPPs in human tissues. Therefore, we performed *ex vivo* tissue-dipping experiments where we studied the distribution of CPP-protein complexes in samples of freshly harvested human tissue material. We used the carcinoma or hyperplasia-containing specimens of the uterus and the cervix, obtained as surgical waste from nine hysterectomies. Our aim was to evaluate the tissue of preference (epithelial versus muscular/connective tissue, carcinoma versus adjacent histologically normal tissue) for two well-studied CPPs, the transportan and the TAT-peptide. We complexed biotinylated CPPs with avidin-β-galactosidase (ABG), which enabled us to apply whole-mount X-gal staining as a robust detection method. Our results demonstrate that both peptides enhanced the tissue distribution of ABG. The enhancing effect of the tested CPPs was more obvious in the normal tissue and in some specimens we detected a striking selectivity of CPP-ABG complexes for the normal tissue. This unexpected finding encourages the evaluation of CPPs as local delivery agents in non-malignant situations, for example in the intrauterine gene therapy of benign gynaecological diseases.

## 1. Introduction

The discovery of the cell-penetrating ability of certain short cationic peptides (cell-penetrating peptides) has fuelled a large number of studies carried out over the last 20 years on both cultured cells and in mice. Despite the potential of cell-penetrating peptides (CPPs) as drug delivery agents, only a few studies have been devoted to the behaviour of CPPs in human tissue. To our knowledge, only the group lead by Roger Tsien has published data about their activatable CPPs in human tumour tissue samples [1,2]. Therefore, we decided to study the distribution of CPP-protein complexes in samples of freshly harvested human tissue material, obtained as surgical waste from hysterectomies. This material is gaining attention as a tissue source for translational research. For example, human fallopian tubes have recently been shown to be an excellent source of adult mesenchymal stem cells, able to be differentiated into muscle, fat, bone and cartilage cell lineages [3]. In addition, several groups have been using surgical waste from hysterectomies to set up *ex vivo* models. For example, cervical tissue from hysterectomies for benign reasons has been used in local delivery studies [4] and cervical tissue from hysterectomies for malign reasons has been used in anti-cancer activity studies [5]. 

Our aim was to evaluate the tissue of preference (epithelial versus muscular/connective tissue, carcinoma versus adjacent histologically normal tissue) for two well-studied CPPs: the transportan and the TAT peptide. We complexed the biotinylated CPPs (bCPPs) with avidin-β-galactosidase (ABG), which enabled us to apply whole-mount X-gal staining as a robust detection method. Tissue samples were an environment for us where CPP-protein complexes had the possibility to “choose” between the different cells. The heterogeneity of cells available in a freshly harvested surgical specimen is most likely very difficult to achieve in cell culture experiments. The TAT-peptide was chosen because it is one of the most widely used CPPs [6] and represents a subgroup of CPPs, which are non-amphipathic CPPs. The transportan, an efficient oligonucleotide delivery enhancer [7,8,9], is an amphipathic CPP. Amphipathic CPPs bear a structural resemblance to cationic amphipathic antimicrobial peptides, several of which have been shown to be selective towards cancer cells both in cultured cells as well as in mice [10]. Therefore, we were eager to elucidate the potential selectivity of the transportan toward human carcinoma tissue.

**Figure 1:**
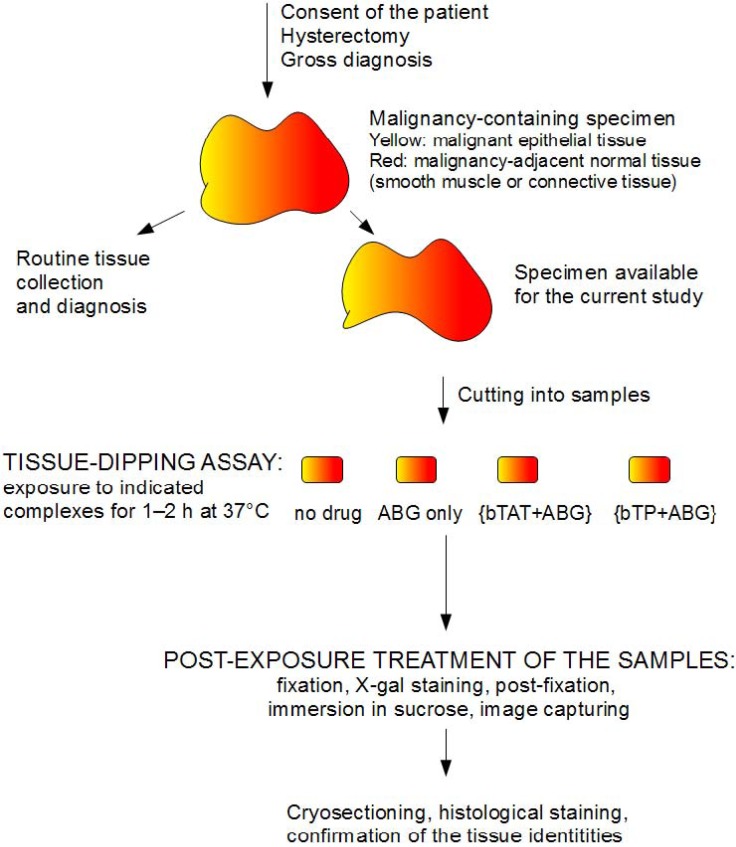
Schematic overview of the experimental setup: specimen processing, tissue-dipping assay and post-exposure treatment of tissue samples.

## 2. Results and Discussion

### 2.1. Hysterectomy Specimens as a Tissue Source

Our aim was to evaluate the possibility that the transportan and the TAT-peptide would differentiate between the different types of tissues, using a tissue-dipping assay. We defined tissue-dipping assay as the exposure of a tissue sample to the biotinylated transportan or the biotinylated TAT-peptide complexed with reporter cargo avidin-β-galactosidase (ABG) over 1–2 h at 37 °C in a well of a 12 or 24-well plate. The optimal specimen for our tissue-dipping assay (Figure 1) had the following characteristics. First, the specimen contained two types of tissue and the visual discrimination of the tissues was possible even for a non-pathologist. Secondly, it was possible to cut the specimen so that each sample of the tissue-dipping assay contained approximately the same volume of each tissue type. 

**Figure 2:**
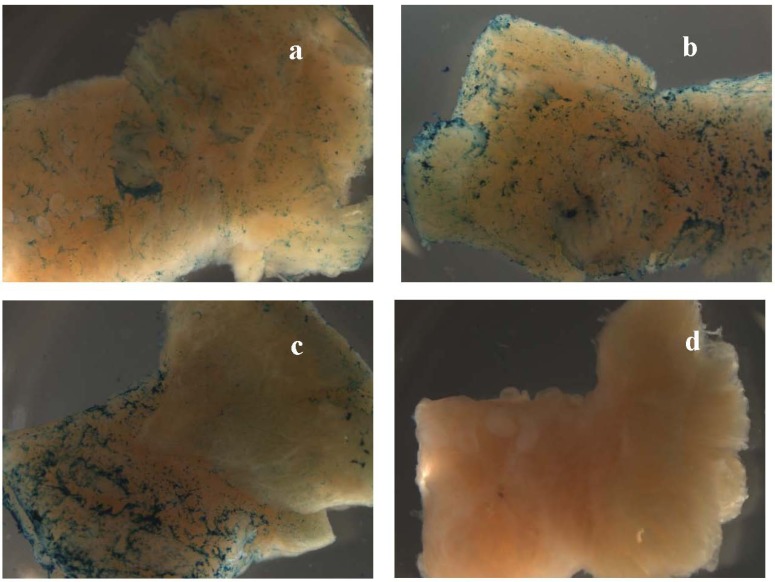
Macrophotographs (at 7.5x magnification) of tissue samples exposed to uncomplexed ABG (a), {bTAT+ABG} (b) and {bTP+ABG} (c) in the experiment abbreviated as “ACE_1” (see Table 1 for details). The tissue samples were from the specimen diagnosed with stage IA adenocarcinoma of the endometrium (ACE). The myometrial parts of samples are directed to the left. The image of the negative control (“no drug”) is in panel d.

These criteria were met best in early-stage adenocarcinomas of the endometrium (ACE). ACE is also known as endometrial/endometrioid adenocarcinoma or simply uterine/endometrial cancer. As ACE is the most common gynaecological malignancy [11], specimens can be obtained relatively often. In stage I ACE specimens, the adenocarcinoma-containing epithelial tissue (endometrium) was readily discriminated from the adjacent normal smooth muscle tissue (myometrium). The discrimination was even easier after the fixation steps as the soft adenocarcinoma areas stood out more strikingly against the firmer myometrium (Figure 2, panel d). The specimens were staged according to the current staging system of the International Federation of Obstetrics and Gynecology (FIGO). In the case of stage IA, the adenocarcinoma was limited to the endometrium (e.g., no invasions to the myometrium). In the case of stage IB, the adenocarcinoma invaded less than half the myometrium. However, the ACE specimens of stage IB were soft and some disaggregation occurred during the exposure step. Therefore, the exposure step was shortened to 1 h in the case of the ACE specimens of stage IB (as specified in Table 1). In addition, because of the softness of the specimen, we did not trim the samples of the stage IB specimen to fit into the wells of the 24-well plate (as we did for the samples of the stage IA specimen). Instead we increased the exposure solution volume to 2 mL and performed the exposure in the wells of the 12-well plate. The histological grade of most of the ACE specimens was grade 1 (G1), meaning that the adenocarcinoma was well-differentiated and had a good prognosis. 

Only two ACE specimens of a higher grade or stage filled the criteria of the optimal specimen. The first specimen had the rare combination of G1 and stage IIIB and the second specimen had the combination of G3 and IB. Histological grade 3 (G3) means that the carcinoma is poorly differentiated and has a poor prognosis. Stage IIIB means that the carcinoma has spread to the vagina but not to lymph nodes or distant sites.

ACE is a disease that predominantly affects post-menopausal women. This means that ideal controls—specimens from age-matched women containing normal endometria in their proliferative phase—do not exist. Unfortunately, the specimens from pre-menopausal women containing endometria in their proliferative phase were not available for this study. Therefore, we used a specimen from a patient who had been diagnosed with hyperplasia of the endometrium (excessive proliferation of the cells of the endometrium). The histological diagnosis of the specimen specified that the hyperplasia was without atypia, indicating a low probability for the development of cancer. The hyperplasia specimen filled the criteria of the optimal specimen: easily discriminated hyperplastic endometrium and adjacent normal myometrium and the possibility to cut the specimen so that each sample contained approximately the same volume of each tissue type. However, hysterectomies are routinely not performed in the case of endometrial hyperplasia, so hyperplasia specimens are rare.

Hysterectomies in patients diagnosed with squamous cell carcinoma of the cervix (SCCC) provided the specimens carrying the combination of squamous cell carcinoma tissue (e.g., epithelial malignant tissue) and the adjacent normal cervical stroma. The cervical stroma is mainly composed of fibrous connective tissue with small amounts elastic fibres and smooth muscle [12]. The histological grade of both SCCC specimens was G3. The first SCCC specimen had stage IVB, meaning that the cancer had spread to distant organs beyond the pelvic area. The patient had also had previous chemotherapy. The second SCCC specimen had stage IB2, meaning that the cancer had grown into the cervix but had not spread anywhere else. This SCCC specimen had very little of the cervical stroma. In summary, the SCCC specimens were not fully optimal for the current study. However, the SCCC specimens were a valuable source of G3-carcinoma tissue and adjacent normal (mainly) connective tissue. 

### 2.2. ABG (Avidin-β-Galactosidase) as a Reporter Cargo in Tissue-Dipping Experiments

**Figure 3:**
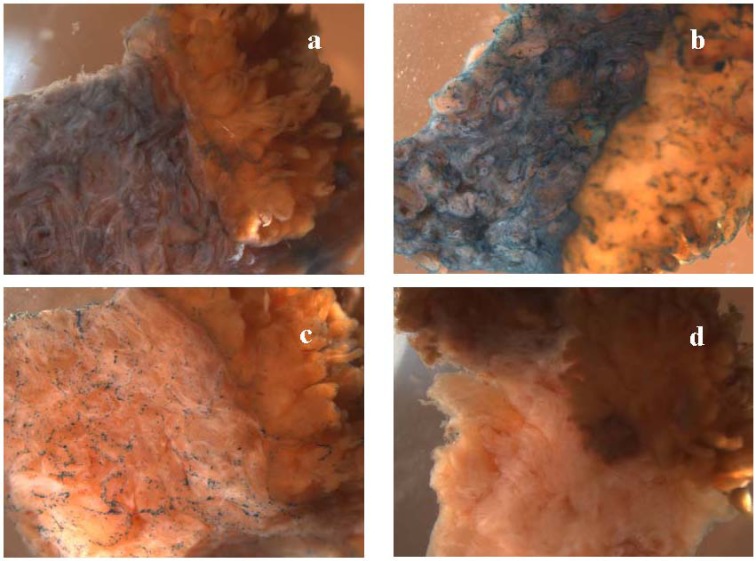
Macrophotographs (at 10x magnification) of tissue samples exposed to 60 μg of uncomplexed ABG (a), to a complex of 60 μg ABG with 5 μM bTP (b) and to a complex of 5 μg ABG with 5 μM bTP (c) in the experiment abbreviated as “SCCC_1” (see Table 1 for details). The tissue samples were from the specimen diagnosed with stage IVB squamous cell carcinoma of the cervix (SCCC). The cervical stromal parts of samples are directed to the left (a, b, c) or down (d). The image of the negative control (“no drug”) is in panel d.

**Figure 4:**
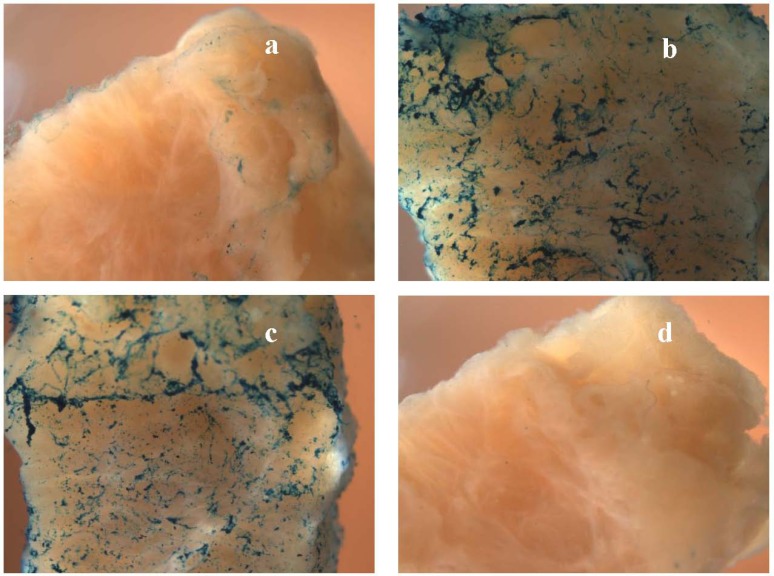
Macrophotographs (at 10x magnification) of tissue samples exposed to uncomplexed ABG (a), {bTAT+ABG} (b) and {bTP+ABG} (c) in the experiment abbreviated as “hyperplasia” (see Table 1 for the details). The tissue samples were from the specimen diagnosed with hyperplasia of the endometrium without atypia. The image of the negative control (“no drug”) is in panel d.

In this study we used ABG as a reporter cargo. In ABG the β-galactosidase from *E. coli* was labelled with avidin from egg white. The avidin part of ABG enabled complex formation with biotinylated CPPs (bCPPs). The β-galactosidase part enabled a robust detection system based on X-gal staining. The X-gal is the most widely used chromogenic substrate for β-galactosidase. The cleavage of the glycosidic linkage in X-gal by β-galactosidase produces colourless indoxyl moieties that are nonenzymatically dimerised and oxidised to halogenated indigo—A stable and insoluble blue compound. Dimerisation and oxidation require the presence of electron acceptors of the proper redox potential. We used the most widely used electron acceptors in the X-gal staining procedure—ferric and ferrous ions. The main concern when using β-galactosidase activity as a part of the reporter cargo is the endogenous β-galactosidase activity in tissues, a well-known problem in mice bearing the *lacZ*-containing transgene [12]. However, we never detected the endogenous β-galactosidase activity when we performed the whole-mount X-gal staining at pH 7.4, optimal for *E. coli* β-galactosidase. In other words, we could not detect the blue indigo colour in our negative controls (“no drug”), or the tissue samples exposed to the protease inhibitor cocktail-containing RPMI medium only (panel d in Figure 2, Figure 3 and Figure 4). 

As expected, endogenous β-galactosidase activity could be readily detected when the pH was lowered to 6.1 (data not shown). The absence of endogenous β-galactosidase activity was the major factor that made us choose ABG as the reporter cargo in our study. In the preliminary experiments we also used fluorescence-based detection. In those experiments we complexed the bCPPs with mouse anti-biotin antibodies and studied the distribution of the complexes by immuno-fluorescence in the cryosections. However, the autofluorescence background was high throughout the visible spectrum, especially in the carcinoma tissue. This complicated the comparison of the distribution of the bCPP-antibody complexes between the carcinoma and the adjacent normal tissue and required extensive image processing. 

In some experiments we noticed the development of green-yellow or brown colour in malignant epithelial tissue. Colour development was more intensive in the G3-carcinoma specimens than in the G1-carcinoma specimens or hyperplastic specimens. The colour development was detectable in all samples of those experiments, e.g., even in the negative control. The most striking example is visualised in Figure 3, panel d. This colour development is most likely due to the production of Prussian green or related products from the ferricyanide and ferrocyanide. The brown colour developed during the staining step and changed to green-yellow when 4% PFA in PBS was used as the post-fixation agent (experiment “ACE_6”, Table 1) and remained brown when glutaraldehyde was used as a post-fixation agent (experiment “SCCC_1”, Table 1 and Figure 4). Whether or not the colour development correlates with the malignancy grade and reflects the redox state of the carcinoma remains to be studied.

**Figure 5:**
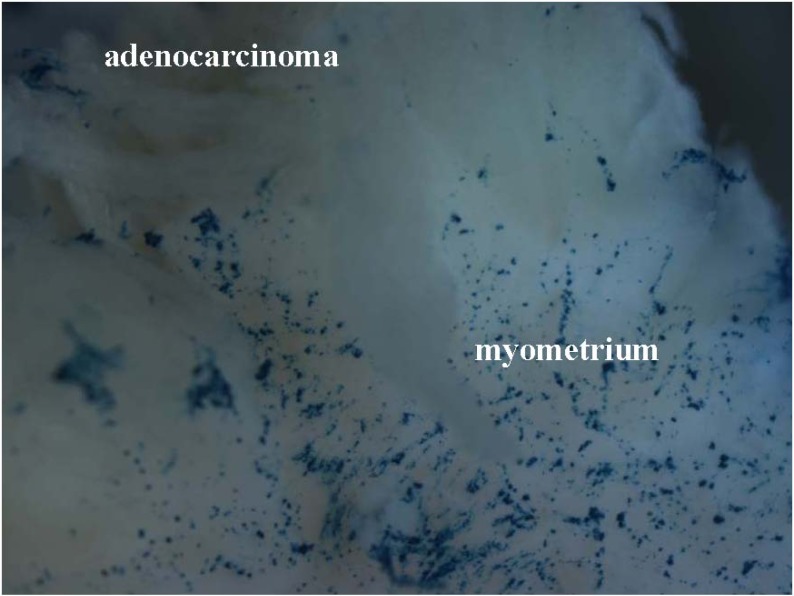
The macrophotograph (at 25x magnification) of the tissue sample exposed to {bTAT+ABG} in the experiment abbreviated as “ACE_4” (see Table 1 for details). The tissue sample was from the specimen diagnosed with stage IB adenocarcinoma of the endometrium (ACE). Note the blue X-gal staining in the myometrium and the absence of the staining in the adenocarcinoma and its invasions into the myometrium.

Whole-mount X-gal staining was readily detectable when the amount of bCPP-complexed ABG in the tissue-dipping assay well was 33–66 μg (0.025–0.1 μM, see Table 1 for details). When only 5 μg of ABG was complexed with bCPP, X-gal staining was remarkably weaker (Figure 3 panel c). The tissue-uptake-enhancing effect of the TAT-peptide and the transportan was readily seen at concentrations of 15–20 μM. The 5 μM bCPP concentration provided readily detectable X-gal staining in experiment “SCCC_1” (Figure 3), but only weak staining in experiments “ACE_3” and “SCCC_2” (see Table 1 for details). Surprisingly, the further lowering of the bCPP concentration to 3.25 μM provided readily detectable X-gal staining in experiments “ACE_4” (Figure 5), “ACE_5” and “ACE_6”. This could be due to the bCPP/ABG molar ratio, which was 50:1 in the experiments with overall weak staining (“ACE_3” and “SCCC_2”) and 55:1 or higher in the rest of the experiments. 

### 2.3. Distribution of {bCPP+ABG} Complexes in Tissue Samples

In every experiment we could readily detect the enhancing effect of the transportan and the TAT-peptide (reflected in the last three columns in Table 1 and in Figure 2, Figure 3, Figure 4, panels a–c). In other words, the X-gal staining intensity of uncomplexed ABG was always lower than the X-gal staining intensity of {bTAT+ABG} and {bTP+ABG}. This was most prominent in the hyperplasia specimen (Figure 4).

**Table 1:** Results from the tissue-dipping experiments.

Specimens	Experiment abbreviation and comments	Exposure solution parameters			Malignancy grade, stage	Character of X-gal staining, Figure number and panel		
		[bCPP] μM	ABG μg	molar ratio bCPP/ABG		ABG	{bTAT+ABG}	{bTP+ABG}
ACE specimens: contained malignant epithelial tissue (malignant endometrium, E) and adjacent histologically normal smooth muscle tissue (normal myometrium, M)	ACE_1	15	60	165:1	G1, stage IA	even Figure 2a	even Figure 2b	M >> E Figure 2c
	ACE_2	15	66	150:1	G1, stage IA	E > M	even	M > E
	ACE_3^§^	5	66	50:1	G1, stage IB	-	even*	even*
	ACE_4^§¥^	3.25	33	65:1	G1, stage IB	-	M >> E Figure 5	even*
	ACE_5^§¥^	3.25	33	65:1	G1, stage IIIB	-	even*	even
	ACE_6^§¥^	3.25	33	65:1	G3, stage IB	-	M > E	M > E
SCCC specimens: contained malignant epithelial tissue (E) and adjacent histologically normal cervical stroma (C)	SCCC_1 post-fixation with glutaraldehyde	5 5	60 5	55:1 661:1	G3, stage IVB previous chemotherapy	C > E Figure 3a	not tested	C >> E Figure 3b-c
	SCCC_2	5	66	50:1	G3, stage IB2	-	even*	even*
Hyperplasia of the endometrium: contained hyperplastic endometrium and adjacent histo-logically normal myometrium	hyperplasia	20	66	200:1	-	- Figure 4a	even Figure 4b	even Figure 4c

ACE: adenocarcinoma of the endometrium; SCCC: squamous cell carcinoma of the cervix;

* denotes weak staining; ^§^ denotes soft speci­men and 1 h exposure; ^¥^ denotes 2 mL exposure solution.

We wanted to exclude the possibility that weak or non-detectable X-gal staining of uncomplexed ABG was due to its adherence to the walls of the wells, thereby diminishing the ABG available for the tissue. Therefore, we performed a quantitative luminescence-based analysis of the residual β-galactosidase activity of the exposure solutions. We found that in the case of the non-detectable X-gal staining in the tissue samples exposed to uncomplexed ABG, the residual β-galactosidase activity value in the exposure solution was the same as the start value (e.g., before the immersion of the tissue sample).

In four out of six ACE specimens, we could detect a difference in the X-gal staining intensities in the same tissue sample when exposed to the {bCPP+ABG} complexes. Surprisingly, it was the normal tissue that had more intensive X-gal staining (last two columns in Table 1). In two ACE specimens we detected a remarkable selectivity of either {bTAT+ABG} or {bTP+ABG} for the normal myometrium. In experiment “ACE_4” we detected a obvious selectivity of the {bTAT+ABG} complex for the normal myometrium and exclusion from the carcinoma area (Figure 5). In experiment “ACE_1” we detected an noticeable selectivity of the {bTP+ABG} complex for the normal myometrium and exclusion from the carcinoma area (Figure 2, panel c). The histological examination of the haematoxylin-eosin stained cryosections of these tissue samples could not point out any differences between the {bTP+ABG}-preferring myometrium and the {bTAT+ABG}-preferring myometrium, as both were histologically normal. The only difference between them was the stage of the adjacent carcinoma: the {bTP+ABG}-preferring myometrium was adjacent to stage IA carcinoma whereas the {bTAT+ABG}-preferring myometrium was adjacent to stage IB carcinoma. One common phenomenon of carcinomas is field cancerisation, which is the occurrence of molecular alterations in the histologically normal tissues surrounding the areas of an overt malignancy [13]. The highest number of markers of field cancerisation is specified for head and neck squamous cell carcinoma, followed by oesophageal cancer [14]. Unfortunately, the field cancerisation of adjacent and distant tissues has not been studied in the case of ACE. In other words, we did not have a set of established markers to evaluate the condition of the myometria of the tissue samples used in this study.

In accordance with the results obtained in the ACE specimens, both CPPs enhanced ABG-uptake in the SCCC specimens. In experiment “SCCC_1” we detected a preferential localisation of {bTP+ABG} complexes in the normal cervical stroma (Figure 3 panel b).

Our second aim was the comparison of the CPP-induced distribution of ABG in different normal tissues (epithelial versus connective versus muscular). Based on X-gal staining, the distribution was enhanced in all normal tissues tested. The quantification of the enhancing effect of CPPs in normal tissue was beyond the scope of this study.

In most specimens used in our study, both tested CPPs showed a preference for histologically normal tissue. We do not currently have an explanation for such selectivity. However, in the light of some recent studies, the lower uptake of CPP-protein complexes in malignant cells is not fully surprising. The *in vitro* uptake of CPP-protein complexes consisting of avidin complexed with bTAT and bTP occurs dominantly via caveolin-mediated endocytosis [15]. The *in vitro* uptake of an albumin-based magnetic resonance contrast medium is also suggested to occur *via* caveolin-mediated endocytosis [16]. When the albumin-based contrast medium was administered intravenously to mice bearing subcutaneous tumours, the histological analysis of the tumours revealed that the contrast medium was internalised by perivascular myofibroblasts and excluded from tumour nodules [17]. Myofibroblasts (also known as cancer-associated fibroblasts) are not malignant cells but are one the most abundant cell types in the tumour stroma [18]. One could conclude that the caveolin-based uptake of the albumin-based contrast medium was more efficient in non-malignant cells than in malignant cells. Whether or not this is the case for the CPP-protein complexes used in our study (e.g., the detailed analysis of their internalisation mechanisms in the presence of both malignant and non-malignant cells) remains to be studied.

We believe that our findings encourage the evaluation of CPPs as local delivery agents in non-malignant situations, for example in the intrauterine gene therapy of benign gynaecological diseases such as leiomyoma. Uterine leiomyomas (also known as uterine fibroids) are the most common benign tumours of the female genital tract [19] and a leading cause of hysterectomy in premenopausal women [20]. The development of a nonsurgical and localised treatment would greatly benefit many women [21]. Uterine leiomyomas are attractive targets for gene therapy because the disease is localised and well circumscribed in the uterus, making ultrasound-guided intratumoural injection simple [22]. Indeed, the adenovirus-mediated delivery of dominant-negative oestrogen receptor genes [21] and herpes simplex virus 1 thymidine kinase genes followed by Ganciclovir treatment [23] shrank the leiomyomas in Eker rats when administered intratumourally. As several CPPs have been shown to enhance adenovirus-mediated transduction [24], the application of the same approach in the context of intrauterine gene therapy is promising.

## 3. Experimental

### 3.1. Peptides

The biotinylated transportan (bTP: GWTLNSAGYLLGK(biotinyl)INLKALAALAKKILamide) was purchased from Inbiolabs (Tallinn, Estonia). The biotinylated TAT-peptide (bTAT: biotinyl-GRK KRRQRRRPPQamide) was synthesised, purified and analysed as previously described [25]. Biotin labelling of both peptides was performed during solid-phase synthesis.

### 3.2. Specimen-Processing

The human tissue materials used in this study were obtained as surgical waste from patients undergoing hysterectomies at the Haematology and Oncology Clinic of the University of Tartu Hospital. This study was approved by the Ethics Review Committee on Human Research of the University of Tartu (protocol numbers 154/3, 20.11.2006 and 179/M-15 16.02.2009) and informed consents were obtained from the patients. The reasons for the hysterectomies were the following: ACE (adenocarcinoma of the endometrium), SCCC (squamous cell carcinoma of the cervix) and hyperplasia of the endometrium. The quality of the surgical specimen was maximised by close collaboration with the operating theatre. In other words, all measures were taken to minimise the time when the specimen sat unattended (a well-known problem in studies involving surgical resection specimens, [26]). Immediately after the surgical resection, the material was examined by a pathologist. Materials were considered suitable for the current study if the carcinomas or hyperplastic tissues were readily distinguishable upon visual inspection. Specimens were in cold RPMI-1640 medium (PAA Laboratories GmbH, Pasching, Austria) during transport to the lab (e.g., until the cutting step; see Figure 1 for the overview of specimen-processing). 

### 3.3. Tissue-Dipping Assay

Samples that could freely move in a well of a 12 or 24-well plate were cut using a Feather S35 microtome blade and submerged in 1 mL of exposure solution. Each tissue sample cut from an ACE specimen contained both malignant epithelial tissue (endometrium) and adjacent normal myometrial tissue (myometrium). Each tissue sample cut from an SCCC specimen contained both malignant epithelial tissue and adjacent normal cervical stroma. Each tissue sample cut from the hyperplasia of the endometrium contained both hyperplastic endometrium and adjacent normal myometrium. The exposure was performed in the wells of a 12 or 24-well plate at 37 ºC on a shaking platform. The wells of the plates were pre-coated with 1% bovine serum albumin solution (in the 1:1 mixture of water and RPMI-1640 medium) in order to diminish the adhesion of the protein to the plastic. The wells were incubated with 2–3 ml of pre-coating solution. After 30 min. the pre-coating solution was aspirated and the wells were considered ready for the exposure step.

The exposure solution was prepared as follows: first the bCPP was complexed with ABG (Sigma-Aldrich Corp. St. Louis, MO, USA) by mixing the appropriate amounts of 1 mM bCPP and ABG (1.25 or 1 mg/ml) and incubating the obtained mixture for 10 min. at room temperature. In the case of biotinylated transportan (bTP), the peptide solution was first treated with L-leucine as described in [27] in order to dissociate the multimers of bTP. The {bCPP+ABG} complexes were diluted with 1 ml of RPMI-1640 medium (2 ml in the case of the tissue samples from the ACE specimens of stage IB) and transferred to the wells of a 24-well plate (12-well plate in the case of the tissue samples from the ACE specimens of stage IB). Then 10 μl of the protease inhibitor cocktail (Sigma-Aldrich Corp) was added in each well, followed by submersion of the tissue sample. The exposure was performed at 37 ºC in a Thermomixer Comfort (Eppendorf AG, Hamburg, Germany) using interval mixing mode (every 2 min., the plate was shaken for 10 seconds at 300 RPM).

### 3.4. Post-Exposure Treatment of Tissue Samples

The samples were washed once with a phosphate-buffered saline (PBS) and then submerged in cold 4% paraformaldehyde (PFA) in PBS and refrigerated for 90 min. The fixative was changed once after 45 min. The fixative aliquots were stored at -20 ºC and always thawed on the day of the experiment. Then the samples were washed several times in a rinsing buffer (100 mM sodium phosphate (pH 7.4), 150 mM NaCl, 2 mM MgCl_2_) and left overnight in the rinsing buffer in the refrigerator. The following day the samples were left to stand at room temperature for an hour, washed once more with the rinsing buffer and then stained for 4 h at 37 ºC in the dark using a Thermomixer Comfort (every 5 min., the plate was shaken for 10 seconds at 300 rpm). The stain solution had the following composition: 100 mM sodium phosphate (pH 7.4), 150 mM NaCl, 2 mM MgCl_2_, 35 mM potassium ferricyanide, 35 mM potassium ferrocyanide, and 1 mg/mL 5-bromo-4-chloro-3-indolyl β-D-galactopyranoside (X-gal). After the staining procedure, the samples were washed several times with PBS and post-fixed in 4% PFA in PBS and refrigerated. Then the samples were washed several times with cold PBS, weighed, immersed in cold 30% sucrose (in PBS with 0.05% sodium azide) and stored in the refrigerator until image recording and cryosectioning. The images of the samples were recorded using an Olympus SZX12 microscope system equipped with an Olympus XC50 camera. 

### 3.5. Cryosectioning

The 25 μM-cryosections were cut using a Slee MNT Cryostat (Slee Medical GmbH, Mainz, Germany) using Feather S35 microtome blades. The optimal temperatures for the cryosectioning of the tissue samples were determined empirically and turned out to be the following: -26 ºC as the chamber temperature and -6 ºC as the specimen temperature. The tissue samples were surrounded by the Tissue-Tek® O.C.T.™ Embedding Compound (Electron Microscopy Sciences, Hatfield, PA, USA) sectioned, applied to Superfrost®Plus slides (Menzel GmbH & Co, Braunschweig, Germany), air-dried and then stored at -20 ºC. Before standard haematoxylin and eosin staining, the slides were thawed, fixed with 4% PFA in PBS (5 min. at room temperature) and then washed with water.

### 3.6. Analysis of the Residual β-Galactosidase Activity in the Exposure Solutions

The 50 μL samples of exposure solutions were mixed with 450 μL of Reaction Lysis Buffer (RLB, Promega) and stored at -20 ºC. On the assay day the samples were further diluted with RLB to obtain the 100-time dilution of the original samples. The 20 μL samples of the final dilution were mixed with 100 μL of the Beta-Glo™ reagent (Promega Biotech AB) in a well of a white 96-well plate and after 30 min, luminescence was recorded in a GENios Plus (Tecan Austria GmbH, Grödig, Austria). The standard curves of ABG ranged from 1 μg/mL to 0.01 μg/mL.

## 4. Conclusions

In this study we evaluated the tissue of preference for two well-studied CPPs—the transportan and the TAT-peptide, complexed with ABG. We described a robust assay based on whole-mount X-gal staining. Our results indicated that both the transportan and the TAT-peptide enhanced the tissue distribution of ABG. In some cases we could even detect a remarkable selectivity of {bTP+ABG} and {bTAT+ABG} for the histologically normal tissue. This unexpected finding encourages the evaluation of CPPs as local delivery agents in non-malignant situations, for example in the intrauterine gene therapy of benign gynaecological diseases.

## Abbreviations

ABG: avidin-β-galactosidase
ACE: adenocarcinoma of the endometrium
bTAT: biotinyl-GRKKRRQRRRPPQamide
{bTAT+ABG}: non-covalent complex between bTAT and ABG
bTP: GWTLNSAGYLLGK(biotinyl) INLKALAALAKKILamide
{bTP+ABG}: non-covalent complex between bTP and ABG
CPP: cell-penetrating peptide
FIGO: International Federation of Obstetrics and Gynecology
G1: grade 1 tumour
G3: grade 3 tumour
TAT-peptide: HIV-1 Tat protein (48–60) amide
o/n: overnight
PBS: phosphate-buffered saline
PFA: paraformaldehyde
RLB: Reaction Lysis Buffer
SCCC: squamous cell carcinoma of the cervix
X-gal: 5-bromo-4-chloro-3-indolyl β-D-galactopyranoside
